# Integrate and work together — compartments as functional units

**DOI:** 10.1007/s00709-022-01778-7

**Published:** 2022-06-03

**Authors:** Peter Nick

**Affiliations:** Botanical Institute, Karlsruhe Institute of Technology, Karlsruhe, Germany

Compartments are seen as core of cell biology — often defined as closed entities, delineated by a contiguous membrane, such that different chemistries that otherwise would be mutually exclusive can proceed in parallel. This classical textbook view is obviously incomplete because prokaryotes should then, per definition, lack compartments. However, it is meanwhile undisputed that also bacteria use compartments (for review see Cornejo et al. 2014), calling for a wider definition, where compartments are functional, rather than merely structural units. It has, thus, become clear that compartments are entities, but not necessarily delineated as separate objects. Nevertheless, they are endowed with a kind of “wholeness”, that is “more than the sum of its parts”. This is not easy to accept. In the words of von Bertalanffy (1950), “the exact scientist therefore is inclined to look at these conceptions with justified mistrust”, for “…these concepts…. are of a vague and somewhat mystical character. Thus, it seems necessary to formulate these conceptions in an exact language”, which was the motivation to coin his famous general system theory. Two contributions to the current issue deal with examples that illustrate that such a systemic approach to compartments as functional units can be fruitful.

The unicellular fungus *Phycomyces blakesleeanus* has been a classical model to study directional growth (so called tropism) in response to directional stimuli, such as light or gravity. Introduced and developed as experimental system by the late Max Delbrück (reviewed in Delbrück 1962), *Phycomyces* became a central model in the application of physical approaches to biological problems complementing Delbrück’s achievements in the establishment of molecular biology. The bending sporangiophore is unicellular but contains many nuclei. Perception of the stimuli and their translation into a response are located in the growing zone in the stalk of the sporangiophore that is connected to a head-like structure that will later generate the mucus with the spores and is termed columella. This columella represents a cytoplasmic unit with the growing zone, and there are neither membranes nor cell walls separating the two regions of the sporangiophore. In their contribution to the current issue, Živanović et al. (2022) address the responses of the columella to auxin growth responses. Despite the intensive research it attracted, *Phycomyces* still has retained some of its secrets. Located in a quite distant and basal clade of the fungi, the Mucorales, this organism exhibits a rapid and efficient photo- and gravitropism, an ability that is otherwise only seen in plants. In plants, tropisms are accompanied by a redistribution of polar auxin transport, raising the question, whether *Phycomyces* has acquired tropism independently, or whether there might be evolutionary ancient homologies between these systems. In their previous work (Živanović et al. 2018), these authors had compared the wild type and the mutant *madC*, which is not able to deploy phototropism and found specific and dose-dependent responses that were also depending on the transport properties of the respective auxin. Moreover, they located *bona-fide* homologues of plant auxin-efflux facilitators and importers in the *Phycomyces* genome, supporting a role of auxin also in the phototropism of this organism. Now, they extend this work by experiments, where the columella is stripped from spores and cell wall and then treated by exogenous auxin. They find a local membrane depolarisation accompanied with inhibition of growth in the spatially separated growing zone, indicative of a signal conveyed from the columella to the growing zone. To get insight into the nature of this signal, they use again a mutant, affected in the activator of the small GTPase Ras. This mutant is altered in auxin sensitivity and membrane depolarisation, reflected in altered response patterns of the growing zone. The authors come up with a model, where the columella participates in signal transduction and communicates with the growing zone by basipetal auxin transport. This looks like a homology of tropisms in higher plants. However, there, the response of a multicellular organ is brought about by different cells. In case of *Phycomyces*, it is the interaction of different regions within a single cell that communicate by signals. These regions are functionally distinct, but not delineated by membranes. Is it concerted signalling that renders these cytoplasmic regions into functional entities? If v. Bertalanffy had known this example, he would have been delighted.

Also, the contribution by Sumiya (2022) deals with an integration of parts into a new whole. The glaucophyte algae *Cyanophora paradoxa* has fascinated evolutionary biologists since decades. These unicellular algae have domesticated so-called cyanelles, remnants of cyanobacteria that have lost their autonomy, although being still not subdued to the level of nuclear rule typical for chloroplasts. The number of cyanelles is low, in most species only two, which means that the division of the host cell and that of the hosted cyanelle must be coupled to avoid that daughter cells exit void of these organelles. In the sister species *C. sudae*, the number of cyanelles has been doubled to four, reporting an episode of interrupted coupling in the past. The authors ask whether the coupling is still active, or whether cell and organelle divide independently, as it is the case in most plants where each cells harbours a large and undefined number of plastids. Using camptothecin, an inhibitor of topoisomerase, arresting the cell cycle in S-phase, the number of cyanelles is shown to double to eight, meaning that the progression to the S-phase deploys a signal that initiates cyanelle division. Conversely, ampicillin, blocking the formation of the cyanelle cell wall, arrests the host cell at the S-G_2_ transition, and cephalexin, blocking the formation of cyanelle septa results in cells that have only one cyanelle, but two nuclei. Thus, the mutual signalling of cell cycle and organelle division known from the two-cyanelle species is retained. Interestingly, it is not mitosis, which is controlled by the division of the cyanelle, but cytokinesis. Overall, the former endosymbiont has lost its autonomy, not only with respect to gene transfer to the nucleus but also with respect to propagation, which can proceed only in response to a signal from the host cell. On the other hand, the host cell has shifted its own propagation under control of a signal from the endosymbiont. Again, mutual signals (probably of inhibitory nature) constitute a new functional entity from hitherto autonomous subsystems.

The General System Theory (v. Bertalanffy 1950) ends with a comparison of a static world of entities with the new world of dynamic systems: “The Greek conception of the world was static, things being considered to be a mirroring of eternal archetypes or ideas. Therefore classification was the central problem in science…. In modern science, dynamic interaction appears to be the central problem in all fields of reality.” The columella of *Phycomyces* and the cyanelles of *Cyanophora* illustrate impressively, how new entities emerge and maintain themselves from dynamic interactions. While the electron microscopic era of cell biology was guided by classification of seemingly static objects such as organelles and compartments, it is getting progressively clear that we need a more physiological approach, where these “objects” are understood as functional units deriving from mutual dynamic signalling.
